# Combined and interaction effect of chlamydia pneumoniae infection and smoking on lung cancer: a case-control study in Southeast China

**DOI:** 10.1186/s12885-020-07418-8

**Published:** 2020-09-22

**Authors:** Xin Xu, Zhiqiang Liu, Weimin Xiong, Minglian Qiu, Shuling Kang, Qiuping Xu, Lin Cai, Fei He

**Affiliations:** 1 Department of Epidemiology and Health Statistics, School of Public Health, Fujian Medical University, Fuzhou, 350108 China; 2 The United Innovation of Mengchao Hepatobiliary Technology Key Laboratory of Fujian Province, Mengchao Hepatobiliary Hospital of Fujian Medical University, Fuzhou, 350025 China; 3 The Liver Center of Fujian Province, Fujian Medical University, Fuzhou, 350025 China; 4 Department of Health and Quarantine, The Xiamen Customs of the People’s Republic of China, Xiamen, 361001 China; 5 Department of Thoracic Surgery, The First Affiliated Hospital of Fujian Medical University, Fuzhou, 350005 China; 6 Fuzhou Center for Disease Control and Prevention, Fuzhou, 350004 China; 7 Department of Preventive Medicine, School of Public Health, Fujian Medical University, Fuzhou, 350108 China; 8 Medical Department, The Affiliated Hospital of Putian University, Putian, 351100 China

**Keywords:** Chlamydia pneumoniae infection, Case-control study, Environmental factors, Lung cancer

## Abstract

**Background:**

This case-control study investigated the role of *Chlamydia pneumoniae* (Cpn) infection in the pathogenesis of lung cancer and the combined and interaction effect of Cpn infection, smoking, and various environmental factors.

**Methods:**

The study comprised 449 lung cancer patients and 512 age- and sex-matched healthy controls. All participants provided a 5 ml fasting peripheral venous blood sample for testing Cpn-specific IgG and IgA by using micro-immunofluorescence. Besides analyzing the associations between Cpn and lung cancer, combined effect analysis, logistic regression, and the Excel table made by Andersson were used to analyze the combined and interaction effects of Cpn and environmental factors on lung cancer.

**Results:**

Compared to those with no evidence of serum Cpn IgA or Cpn IgG, those with both Cpn IgG+ and IgA+ had 2.00 times the risk (95% CI: 1.34–3.00) of developing lung cancer. Cpn IgG+ or IgA+ was associated with a significantly increased risk of lung cancer among smokers; the adjusted odds ratio (OR) was 1.79 (95% CI: 1.10–2.91) and 2.27 (95% CI: 1.38–3.72), respectively. Those exposed to passive smoking with Cpn IgG+ or IgA+ also showed an increased risk of lung cancer; the adjusted OR was 1.82 (95% CI: 1.20–2.77) or 1.87 (95% CI: 1.22–2.87), respectively. Similar results were also observed among alcohol drinkers. Multiplicative and additive interactions were not observed between Cpn infection and environmental factors. The combined effects of Cpn IgG+ or IgA+ with smoking, passive smoking, and family history of cancer on lung cancer were determined.

**Conclusion:**

Cpn infection is potentially associated with primary lung cancer in the Chinese Han population and has combined effects with smoking, passive smoking, and family history of cancer.

## Background

Lung cancer is the most commonly diagnosed cancer (11.6% of all cases) and the leading cause of cancer death (18.4% of all cancer deaths) worldwide, with greater than 80% of lung cancers in western populations attributed to smoking [1]. Recently, studies on other causes of lung cancer, such as infections and respiratory diseases, have been increasing in number [2].

Infection is the third leading cause of cancer worldwide [3]. Chronic inflammation resulting from various persistent infections is known to place a person at risk for malignancy. The etiologic role of chronic lung inflammation in lung cancer development has been established [4, 5]. Moreover, chronic lung infections can increase lung cancer risk independently and in conjunction with tobacco smoke exposure [4]. Chlamydia species, including *Chlamydia pneumoniae* (Cpn), *Chlamydia trachomatis* (Ctr), and *Chlamydia psittaci* (Cps), can also cause persistent infections and chronic inflammation, which may play an essential role in lung cancer pathogenesis. Cpn can cause pneumonia and other respiratory infections, and repeated or prolonged exposure to Chlamydia antigens may cause chronic obstructive pulmonary disease, asthma, and lung cancer. Several studies have examined the association between Cpn infection and lung cancer [6–16] but failed to identify the combined and interaction effect of Cpn infection with environmental factors, which is of great value.

This study aimed to evaluate the role of Cpn infection in the pathogenesis of lung cancer and to investigate the combined and interaction effect of Cpn infection and environmental factors on lung cancer.

## Methods

### Cases and controls

Lung cancer cases were identified from the Department of Thoracic Surgery and Respiratory Medicine of The First Affiliated Hospital of Fujian Medical University, Fujian Medical University Union Hospital, and Fuzhou General Hospital of Nanjing Military Command between December 2006 and December 2016. Inclusion criteria: (1) newly diagnosed primary lung cancer by fiberoptic bronchoscopy or histopathologic evaluation, and (2) lived in the Fujian province of China for more than 10 years. Exclusion criteria: (1) pathologic diagnosis of lung inflammation, benign lesion, or secondary lung cancer, and (2) could not answer the study questions.

Controls were frequency matched based on the age and sex of cases. During the same study period, healthy community dwellers were selected for the control group. Inclusion criteria: (1) lived in Fujian province for more than 10 years, (2) no history of tumor, and (3) no family member participated as a case of this study. In total, 449 lung cancer cases and 512 healthy controls were included in this study. The participation rate was 97% for patients and 90% for control subjects. The Institutional Review Board of Fujian Medical University (Fuzhou, China) approved this study, and all participants signed informed consent forms.

### Survey content and variables

This is an ongoing case-control study and the detail of the questionnaire had been published previously [17]. All epidemiological data were obtained through face-to-face interviews using a standardized questionnaire, which collected information on baseline demographic characteristics, body mass index (BMI), smoking, passive smoking, alcohol consumption, tea drinking, history of lung diseases, family history of cancer, occupational physical activity, physical exercise, cooking oil fume exposure, and pollution near the residence.

Smoking was defined as having smoked more than 100 cigarettes in their lifetime. Passive smoking was defined as non-smokers who were exposed to inhaled cigarette smoke or exhaled smoke more than once per day for more than 15 min per day. Alcohol consumption was defined as drinking at least one alcoholic beverage per week for more than 6 months, regardless of alcoholic drink type. Drinking tea was defined as consuming at least one cup per week for more than 6 months. A family history of cancer was defined as the occurrence of a malignant tumor in first-degree or second-degree relatives. Occupational physical activity was rated as low, moderate, or high intensity, following the Reference Standard of Labor Intensity recommended by the Chinese Nutrition Society in 2000 [18]. Participants were asked about fumes in their kitchens during cooking for evaluating cooking oil fume exposure.

Before the survey, we trained investigators strictly. Furthermore, the investigators combined their professional knowledge to make a more objective evaluation of the quality of the questionnaire based on the respondents’ answers, methods, and attitudes. After the survey, 10% of the questionnaire was randomly rechecked to verify the authenticity of the survey data.

### Experimental methods

All cases and controls provided a 5 ml fasting peripheral venous blood sample, using non-anticoagulation vacuum blood collection tubes. Samples were immediately processed by centrifugation at 2000 rpm for 10 min, followed by serum separation and storage at − 80 °C.

**Fig. 1:**
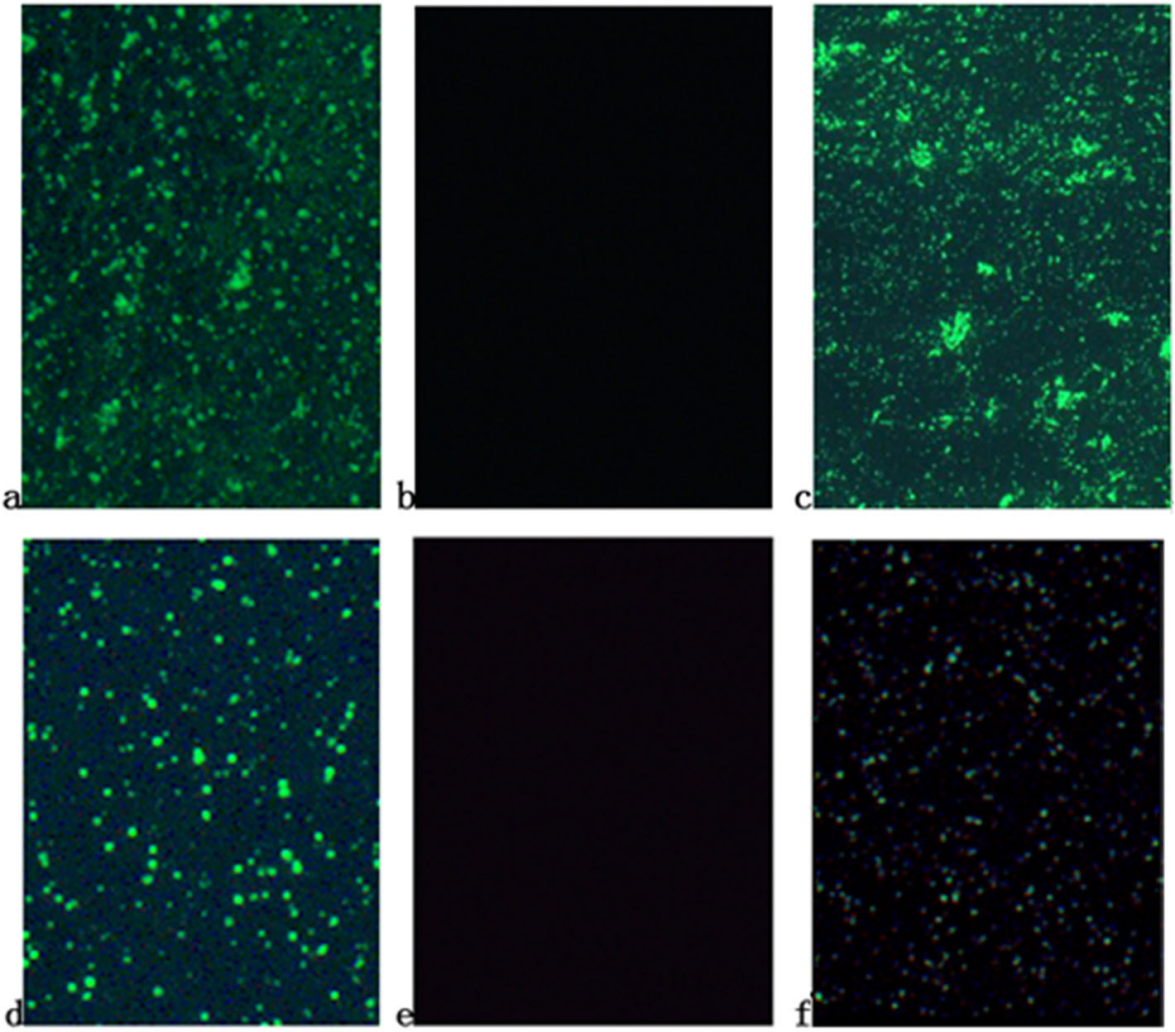
The detecting results of serum Cpn IgG and IgA by MIF method under the microscope (× 400 times. **a**: IgG positive control; **b**: IgG negative control; **c**: IgG positive specimens; **d**: IgA positive control; **e**: IgA negative control; **f**: IgA positive)

Cpn-specific IgG and IgA were tested using a micro-immunofluorescence (MIF) kit (Chlamydia IgG SeroFIA kit and Chlamydia IgA SeroFIA kit, DADE Behring, Savyon Diagnostics, Israel). Positive chlamydia controls produced a moderate apple-green fluorescent color, whereas negative controls did not fluoresce. A positive result for the presence of chlamydia showed a moderately dispersed apple-green fluorescent color; strongly positive results had an intensely glaring apple-green fluorescent color. No fluorescence of any color or a dark background indicated no chlamydial morphology. Although serum Chlamydia IgG and IgA antibody detection are the accepted diagnostic tests for Cpn, MIF test results are subjectively read with the naked eye. To minimize bias, two individuals conducted the experiment: one skilled technician conducted the preliminary experiment, and the second person conducted a blind interpretation of the results. For quality control, 10% of samples were randomly selected for retesting (Fig. 1).

### Statistical analysis

The Chi-square test was used to compare general characteristics for cases and controls. Stratified analysis and unconditional logistic regression were performed to calculate odds ratios (ORs) and 95% confidence intervals (CIs) for chlamydia infection and lung cancer risk. The combined effects and multiplication interaction were analyzed by crossover analysis and logistic regression. The method developed by Andersson [19] was used to evaluate the additive interaction, including the relative excess risk of interaction (RERI), attributed proportion of interaction (API), the synergy index (S), and their 95% CIs. If there is no additive interaction, the 95% CIs of the RERI and API each contain 0, and the 95% CI of S contains 1. The SPSS 24.0 software package (IBM Corporation, Armonk, New York, USA) was used. All *P* values were based on a two-sided test with an α of 0.05.

## Results

### Participant characteristics

A total of 961 patients were enrolled in this study, including 449 cases and 512 controls. There were no baseline differences between groups concerning sex, age, ethnicity, marital status, tea-drinking, decoration within 10 years, and ventilation status (*P* > 0.05). However, cases and controls did differ concerning educational level, occupation, BMI, smoking, passive smoking, alcohol consumption, history of lung diseases, history of other diseases, family history of cancer, occupational physical activity, physical exercise, cooking oil fume exposure, and pollution near the residence (*P* < 0.05). Of the 449 cases of lung cancer, there were 277 (61.7%) with lung adenocarcinomas, 96 (21.4%) with squamous cell carcinoma, 38 (8.5%) with small cell carcinoma, 7 (1.6%) with adenosquamous carcinoma, 2 (0.4%) with large cell carcinoma, and 29 (6.4%) with other types (Table 1).

**Table 1:** Subject characteristics by case and control groups

Variables	Cases N (%) (*N* = 449)	Controls N (%) (*N* = 512)	*χ*^*2*^	*P*
Age (years)			0.194	0.979
< 45	39 (8.7)	43 (8.4)		
45 ~	197 (43.9)	229 (44.7)		
60 ~	192 (42.7)	214 (41.8)		
≥ 75	21 (4.7)	26 (5.1)		
Sex			3.290	0.070
Male	293 (65.3)	305 (59.6)		
Female	156 (34.7)	207 (40.4)		
Ethnicity			0.274	0.601
Han	443 (98.7)	507 (99.0)		
Others	6 (1.3)	5 (1.0)		
Education			90.131	< 0.001
Primary school or less	231 (51.4)	145 (28.3)		
High school	174 (38.8)	199 (38.9)		
College or higher	44 (9.8)	168 (32.8)		
Marital status			0.204	0.651
Married	429 (95.5)	486 (94.9)		
Unmarried or others	20 (4.5)	26 (5.1)		
Occupation			75.731	< 0.001
Agriculture, forestry, animal husbandry and fishery personnel	138 (30.7)	80 (15.6)		
Production transport workers	109 (24.3)	89 (17.4)		
Enterprises and institutions personnel	123 (27.4)	260 (50.8)		
Business service personnel	33 (7.4)	58 (11.3)		
Unemployed or others	46 (10.2)	25 (4.9)		
BMI (kg/m^2^)			11.815	0.003
< 18.5	32 (7.1)	20 (3.9)		
18.5 ~	278 (61.9)	286 (55.9)		
≥ 24.0	139 (31.0)	206 (40.2)		
Smoking			68.114	< 0.001
No	188 (41.9)	350 (68.4)		
Yes	261 (58.1)	162 (31.6)		
Passive smoking			48.699	< 0.001
No	131 (29.2)	263 (51.4)		
Yes	318 (70.8)	249 (48.6)		
Alcohol consumption			13.787	< 0.001
No	308 (68.6)	405 (79.1)		
Yes	141 (31.4)	107 (20.9)		
Tea drinking			1.255	0.263
No	239 (53.2)	254 (49.6)		
Yes	210 (46.8)	258 (50.4)		
History of lung diseases			18.235	< 0.001
No	381 (84.9)	478 (93.4)		
Yes	68 (15.1)	34 (6.6)		
History of other diseases			5.326	0.021
No	264 (58.8)	338 (66.0)		
Yes	185 (41.2)	174 (34.0)		
Family history of cancer			5.070	0.024
No	336 (74.8)	414 (80.9)		
Yes	113 (25.2)	98 (19.1)		
Occupational physical activity			52.066	< 0.001
Light	144 (32.1)	278 (54.3)		
Median	169 (37.6)	150 (29.3)		
Heavy	136 (30.3)	84 (16.4)		
Physical exercise			62.254	< 0.001
Not often	316 (70.4)	231 (45.1)		
Usually	133 (29.6)	281 (54.9)		
Cooking oil fumes			31.925	< 0.001
No	84 (18.7)	167 (32.6)		
Few	231 (51.4)	241 (47.1)		
Some	106 (23.6)	93 (18.2)		
Heavy	28 (6.2)	11 (2.1)		
Decoration within ten years			0.646	0.421
No	278 (61.9)	304 (59.4)		
Yes	171 (38.1)	208 (40.6)		
Pollution near the residence			27.114	< 0.001
No	368 (82.0)	476 (93.0)		
Yes	81 (18.0)	36 (7.0)		
Ventilation status			4.272	0.118
Bad	11 (2.4)	8 (1.6)		
General	48 (10.7)	38 (7.4)		
Well	390 (86.9)	466 (91.0)		
Pathological types			–	–
Adenocarcinoma	277 (61.7)	–		
Squamous cell carcinoma	96 (21.4)	–		
Adenosquamous carcinoma	7 (1.6)	–		
Large cell carcinoma	2 (0.4)	–		
Small cell carcinoma	38 (8.5)	–		
Others	29 (6.4)	–		

### Chlamydia infection and lung cancer

The association between Chlamydia infection and lung cancer can be found in Table 2. Patients with serum Cpn IgG showed significantly increased lung cancer risk (OR = 1.42; 95% CI = 1.02–1.96). Those with serum Cpn IgA showed significantly increased lung cancer risk (OR = 1.73; 95% CI = 1.25–2.38). Both Cpn IgG+ and IgA+ were statistically associated with an increased lung cancer risk (OR = 2.00; 95% CI = 1.34–3.00). No relationships between other Chlamydia infections (Ctr and Cps) and lung cancer were observed.

**Table 2:** The association between Chlamydia infection and lung cancer

Variables	Cases N (%)	Controls N (%)	OR(95%CI)	OR(95%CI)^a^
Cpn				
Cpn IgG				
-(< 1:64)	121 (26.9)	185 (36.1)	1.00	1.00
+ (≥1:64)	328 (73.1)	327 (63.9)	1.53 (1.16–2.02)	1.42 (1.02–1.96)
Cpn IgA				
-(< 1:32)	265 (59.0)	376 (73.4)	1.00	1.00
+ (≥1:32)	184 (41.0)	136 (26.6)	1.92 (1.46–2.52)	1.73 (1.25–2.38)
Cpn IgG or IgA				
both-	105 (23.4)	161 (31.4)	1.00	1.00
single+	176 (39.2)	239 (46.7)	1.13 (0.83–1.55)	1.07 (0.74–1.54)
both+	168 (37.4)	112 (21.9)	2.30 (1.63–3.24)	2.00 (1.34–3.00)
Ctr				
Ctr IgG				
-(< 1:64)	376 (83.7)	447 (87.3)	1.00	1.00
+ (≥1:64)	73 (16.3)	65 (12.7)	1.34 (0.93–1.92)	1.04 (0.69–1.58)
Ctr IgA				
-(< 1:32)	443 (98.7)	509 (99.4)	1.00	1.00
+ (≥1:32)	6 (1.3)	3 (0.6)	2.30 (0.57–9.24)	1.32 (0.30–5.86)
Ctr IgG or IgA				
both-	373 (83.1)	446 (87.1)	1.00	1.00
single+	73 (16.3)	64 (12.5)	1.36 (0.95–1.96)	1.06 (0.69–1.61)
both+	3 (0.6)	2 (0.4)	1.79 (0.30–10.79)	1.12 (0.17–7.58)
Cps				
Cps IgG				
-(< 1:64)	406 (90.4)	481 (93.9)	1.00	1.00
+ (≥1:64)	43 (9.6)	31 (6.1)	1.64 (1.02–2.66)	1.28 (0.73–2.22)
Cps IgA				
-(< 1:32)	444 (98.9)	508 (99.2)	1.00	1.00
+ (≥1:32)	5 (1.1)	4 (0.8)	1.43 (0.38–5.36)	1.82 (0.41–8.10)
Cps IgG or IgA				
both-	404 (90.0)	478 (93.4)	1.00	1.00
single+	42 (9.4)	33 (6.4)	1.51 (0.94–2.42)	1.18 (0.68–2.05)
both+	3 (0.6)	1 (0.2)	3.55 (0.37–34.26)	4.02 (0.36–45.59)

^a^Adjusted by age, sex, education, occupation, BMI, smoking, passive smoking, alcohol consumption, history of lung diseases, history of other diseases, family history of cancer, occupational physical activity, physical exercise, cooking oil fumes and pollution near the residence

**Fig. 2:**
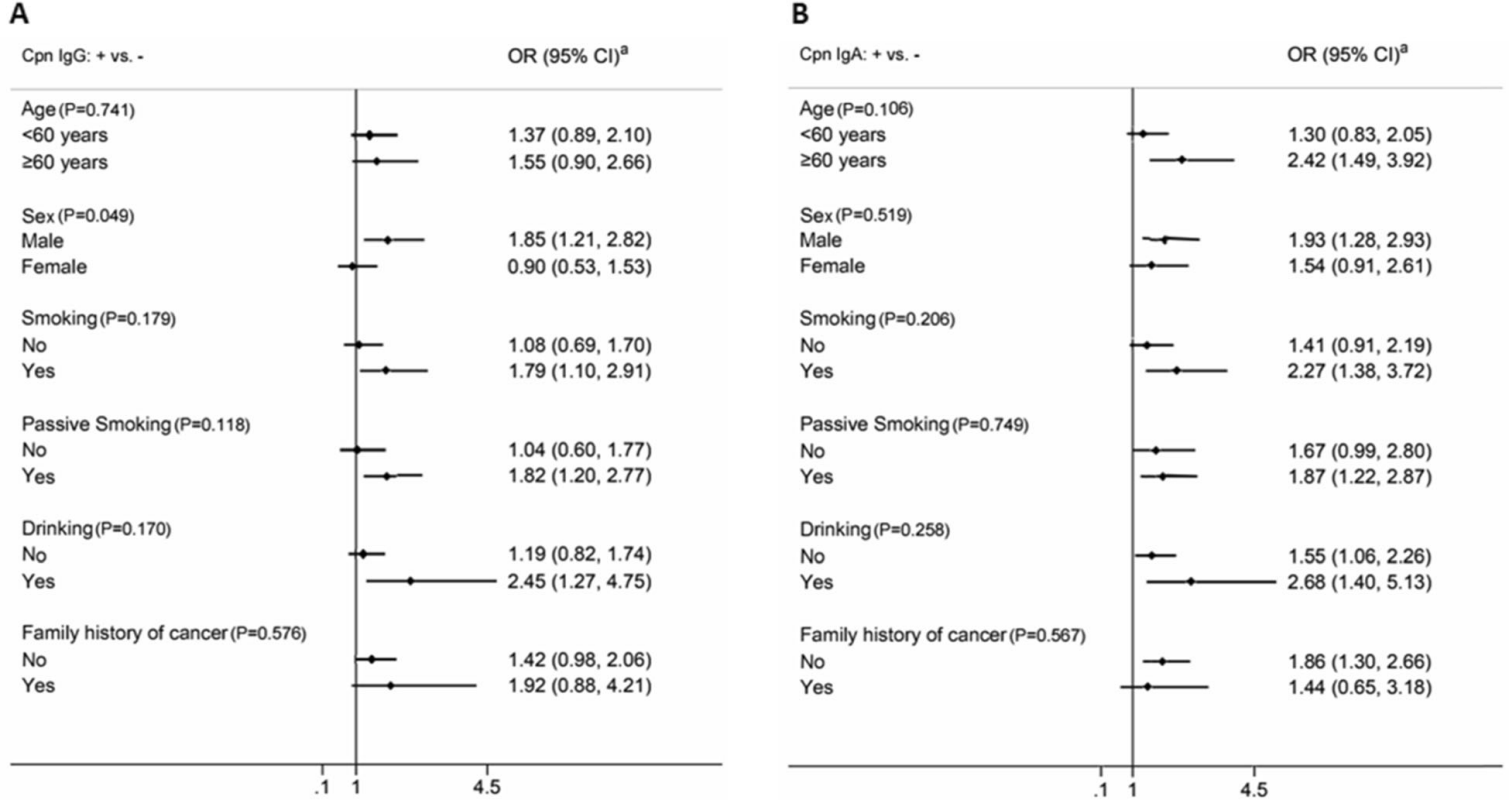
Stratified analysis of the association between Chlamydia pneumoniae infection and the risk of lung cancer. **a** and **b** are the results of IgG and IgA respectively. ^a^Adjusted by age, sex, education, occupation, BMI, smoking, passive smoking, alcohol consumption, history of lung diseases, history of other diseases, family history of cancer, occupational physical activity, physical exercise, cooking oil fumes and pollution near the residence

Stratified analyses were carried out by age, sex, smoking, passive smoking, drinking, and family history of cancer. Adjustment was made for demographic characteristics and relevant factors. The effect between Cpn lgG+ and lung cancer was modified by sex (*P* = 0.049). Among men, those with serum Cpn IgG+ were 1.85 times as likely (95% CI = 1.21–2.82) to develop lung cancer. However, among women, the adjusted OR was 0.90 (95% CI = 0.53–1.53). Among men, those with serum Cpn IgA+ were 1.93 times as likely (95% CI = 1.28–2.93) to develop lung cancer. Among women, the adjusted OR was 1.54 (95% CI = 0.91–2.61). Those aged 60 years and older with Cpn IgA were 2.42 times more likely to develop lung cancer (95% CI = 1.49–3.92). Similarly, among smokers, the risk of developing lung cancer was 1.79 times higher if IgG was positive (95% CI = 1.10–2.91) and 2.27 times higher if IgA was positive (95% CI = 1.38–3.72). The lung cancer risk of passive smokers was 1.82 times higher if IgG was positive (95% CI = 1.20–2.77) and 1.87 times higher if IgA was positive (95% CI = 1.22–2.87). Among alcohol drinkers, those with Cpn IgG+ were 2.45 times as likely to develop lung cancer (95% CI = 1.27–4.75), and those with Cpn IgA+ were 2.68 times as likely to develop lung cancer (95% CI = 1.40–5.13) (Fig. 2).

### Combined and interaction effects of Cpn IgG or IgA and environmental factors

After adjustment for possible confounding factors, the results showed that multiplicative and additive interactions were not observed between Cpn infection and environmental factors. However, the combined effects of Cpn IgG+ or IgA+ and smoking, passive smoking, and family history of cancer on lung cancer were determined (Supplemental Table 1).

## Discussion

The results of this study showed that Cpn infection was associated with the risk of lung cancer. Patients with both serum Cpn IgG+ and IgA+ had 2.00 times the risk of developing lung cancer. The stratified analysis showed that smokers or drinkers with Cpn IgG+ or IgA+ were more likely to develop lung cancer. Additionally, Cpn IgG and IgA each had a combined effect on smoking, passive smoking, and family history of cancer.

Our results were consistent with the results of other studies. Several studies [6, 20, 21] showed that Cpn infection is associated with a higher risk of lung cancer. Furthermore, other studies [6–8] showed ORs of 1.2 to 2.8 after adjusting for smoking status, indicating that chronic Cpn infection is an independent risk factor for lung cancer. Several case-control studies showed that Cpn infection increased the risk of lung cancer development [10–13, 16] but failed to show a correlation between serum Cpn antibodies and cancer risk [14, 15, 22].

Although it is unclear how Cpn infection would induce or cause lung cancer, the process may involve chronic inflammation. Chronic Cpn infections may prolong inflammatory mediator stimulation to increase cell necrosis, apoptosis, and mitosis. Thus, the relationship between Cpn infection and lung cancer seems biologically plausible. Furthermore, Cpn proteins have been shown to trigger lung cancer growth potential by altering host cellular replication, transcription, and DNA damage repair [23]. During tissue repair, active cellular splitting can result in the occurrence, accumulation, and fixation of mutations, deletions, ectoplasias, and amplifications; these changes increase the risk of malignant transformation at the site of infection [24]. Furthermore, cellular experiments also showed that Cpn infection could transform mesothelial cells, which in turn could increase lung cancer risk [25]. Researchers have also established a Cpn infection-induced lung cancer model in rats [26].

Cpn infections are common among specific patient subgroups, particularly young people [6, 11], men [12, 13], and smokers [6, 8, 11]. Furthermore, the relationship between Cpn infection and lung cancer risk may vary when combined with environmental factors (e.g., age, sex, and smoking history). Among patients aged 60 years and older in our study, the association between Cpn IgA and lung cancer was statistically significant. This may be due to the increase of Cpn infection with age. In addition, a significant association was found between Cpn IgA or IgG and lung cancer among males and smokers. The OR values among smokers in our study were consistent with the results of other studies [8, 11]. The current study also suggested that passive smokers with Cpn infection had a higher risk of lung cancer. The same carcinogens in cigarette smoke may induce lung cancer in people with a history of smoking and passive smoking [27–29]. Reactive nitrogen and oxygen species (RNOS) produced by smoking can activate NF-κB to promote the expression of inflammatory genes and directly or indirectly activate the production of inflammatory mediators through the regulation of various protein modifications and degradation [30]. Therefore, Cpn combined with smoking may promote lung cancer via elevated levels of inflammatory factors. Although many studies have indicated that smoking might induce lung cancer by aggravating lung inflammation [30, 31], further research on the underlying mechanisms of Cpn infection in the pathogenesis of lung cancer is still required.

Moreover, the current study also showed, for the first time, that Cpn IgG and IgA were more closely associated with lung cancer among alcohol drinkers. Alcohol exposure reduces airway mucociliary clearance through the progressive desensitization of ciliary response. As a result, this important innate primary defense mechanism is weakened. Chronic alcohol exposure also alters the adaptative immune response to pathogens and leads to an inflammatory response [32]. Therefore, Cpn combined with alcohol drinking may also promote lung cancer via elevated levels of inflammatory factors. Furthermore, the combined effects of Cpn IgG+ or IgA+ and family history of cancer on lung cancer were found. He et al. [33] proposed that non-small cell lung cancer (NSCLC) patients with a family history of cancer, especially a family history of lung cancer, might have a significantly higher incidence of epidermal growth factor receptor (EGFR) activating mutation. EGFR is an important predictive biomarker of EGFR tyrosine kinase inhibitors (TKIs) in NSCLC. Moreover, Cpn proteins have been shown to trigger lung cancer growth potential by DNA damage repair [23]. Therefore, Cpn infection might combine with a family history of cancer to induce lung cancer by mutation. However, further studies are warranted to confirm the results and explore the role of family history of cancer.

In this study, serum Cpn IgG and IgA were detected by MIF, which is the standard for serologic detection of Chlamydia infection. However, the use of MIF is limited by its subjectivity and reproducibility [34]. Therefore, our experiment was conducted by two different people. A skilled technician performed the preliminary experiment, and the second person conducted a blind interpretation of the results. Furthermore, 10% of the samples were randomly selected for retesting. Previously published studies have had varying definitions for “chronic” chlamydial infection. For example, one study [6] used a combination of specific IgA titers (1:16 or higher) and immune complex titers (1:4 or greater), whereas others have used IgA titers of 1:64 or higher [10] or IgG titers of 1:512 or higher [12–14]. Moreover, in several studies [7, 8, 11, 35, 36] IgG antibody titers of 1:16 or more were considered as evidence of past or present Cpn infection, whereas IgA antibody titers of 1:16 or more were considered to indicate chronic infection. Thus, IgG and IgA antibody detection were used to explore the relationship between Chlamydia and lung cancer in the current study.

The present study is the most extensive retrospective case-control study to evaluate the role of Cpn in lung cancer pathogenesis. Meanwhile, stratification and multivariate analysis were used to identify possible effect modifiers associated with Cpn and lung cancer. However, several potential limitations in this study should be considered. First, there were some unavoidable selection and recall biases. Second, it is difficult to explore the causal inference between Cpn infection and lung cancer when the blood for the study is collected after the cancer diagnosis to determine Cpn infection status. Third, our results may underestimate the effect of the association between Cpn infection and lung cancer due to non-disaggregated misclassification bias caused by the pre-selected criteria for determining chlamydial infection. Finally, although Cpn IgG+ means patients have at some point had a Cpn infection, Cpn IgA+ only means patients have present or chronic Cpn infection because of the short half-life of Cpn IgA. Despite these limitations, our findings are biologically plausible. Studies have suggested that higher infection rates in patients with cancer are often caused by the immunosuppressive effects of cancers [6]. However, studies in which serum was collected before lung cancer diagnosis showed that the association between serum Cpn and lung cancer still existed when blood samples obtained 1 to 5 years before diagnosis were excluded, suggesting that Cpn infection pre-dated the cancer diagnosis [8].

## Conclusion

In conclusion, our results show that Cpn infection might be an independent risk factor for lung cancer and it has combined effects with smoking, passive smoking, and a family history of cancer. However, in order to make causal inferences about Cpn infection and lung cancer, well-designed cohort studies and randomized controlled trials are needed to minimize the effect of disease on antibody titers, reduce selection bias, and better adjust for potential confounders. Modifications of these studies would allow tests to clarify the pathogenic role of Cpn infection in lung cancer.

## Supplementary information

**Additional file 1.**

## Data Availability

The datasets used and/or analyzed during the current study are available from the corresponding author on reasonable request.

## Abbreviations

API: Attributed proportion of interaction
BMI: Body mass index
Cpn: Chlamydia pneumonia
Ctr: Chlamydia trachomatis
Cps: Chlamydia psittaci
CIs: Confidence intervals
EGFR: Epidermal growth factor receptor
MIF: Micro-immunofluorescence
ORs: Odds ratios
RERI: Relative excess risk of interaction
RNOS: Reactive nitrogen and oxygen species
S: The synergy index
TKIs: Tyrosine kinase inhibitors
