# Supplementary material for: Combined and interaction effect of chlamydia pneumoniae infection and smoking on lung cancer: a case-control study in Southeast China
Source: BMC Cancer. 2020 Sep 22;20:903. doi: 10.1186/s12885-020-07418-8 (PMC7510273; doi:10.1186/s12885-020-07418-8)
Supplement: Supplementary file 1 — Additional file 1. [file 12885_2020_7418_MOESM1_ESM.docx]

**Table S1** The combined and interaction effects of Cpn IgG or IgA and other factors

| Variables | | | Cases N (%) | | Controls N (%) | | OR(95%CI)^a^ |
| --- | --- | --- | --- | --- | --- | --- | --- |
| Cpn IgG | Smoking |  | |  | |  | |
| - | No | 57(12.7) | | 123(24.0) | | 1.00 | |
| - | Yes | 64(14.3) | | 62(12.1) | | 2.45(1.29-4.67) | |
| + | No | 131(29.2) | | 227(44.4) | | 1.08(0.70-1.67) | |
| + | Yes | 197(43.8) | | 100(19.5) | | 4.33(2.43-7.72) | |
| Cpn IgG× Smoking | | |  | |  | | 0.63(0.33-1.23) |
| *RERI*(95%*CI*) | | |  | |  | | 1.81(0.08-3.53) |
| *API*(95%*CI*) | | |  | |  | | 0.42(0.12-0.71) |
| *S*(95%*CI*) | | |  | |  | | 2.19(0.95-5.05) |
| Cpn IgA | Smoking |  | |  | |  | |
| - | No | 123(27.4) | | 250(48.8) | | 1.00 | |
| - | Yes | 142(31.6) | | 126(24.6) | | 2.94(1.75-4.93) | |
| + | No | 65(14.5) | | 100(19.5) | | 1.31(0.85-2.02) | |
| + | Yes | 119(26.5) | | 36(7.0) | | 6.26(3.43-11.45) | |
| Cpn IgA× Smoking | | |  | |  | | 0.62(0.32-1.21) |
| *RERI*(95%*CI*) | | |  | |  | | 3.02(-0.03-6.06) |
| *API(95%CI)* | | |  | |  | | 0.48(0.22-0.75) |
| *S*(95%*CI*) | | |  | |  | | 2.34(1.20-4.56) |
| Cpn IgG | Passive smoking |  | |  | |  | |
| - | No | 36(8.0) | | 83(16.2) | | 1.00 | |
| - | Yes | 85(18.9) | | 102(19.9) | | 1.10(0.63-1.94) | |
| + | No | 95(21.2) | | 180(35.2) | | 0.86(0.51-1.47) | |
| + | Yes | 233(51.9) | | 147(28.7) | | 2.06(1.24-3.43) | |
| Cpn IgG× Passive smoking | | |  | |  | | 0.54(0.27-1.07) |
| *RERI*(95%*CI*) | | |  | |  | | 1.10(0.44-1.75) |
| *API*(95%*CI*) | | |  | |  | | 0.53(0.21-0.86) |
| *S*(95%*CI*) | | |  | |  | | -29.14(-) |
| Cpn IgA | Passive smoking |  | |  | |  | |
| - | No | 80(17.8) | | 188(36.7) | | 1.00 | |
| - | Yes | 185(41.2) | | 188(36.7) | | 1.72(1.18-2.53) | |
| + | No | 51(11.4) | | 75(14.6) | | 1.48(0.89-2.46) | |
| + | Yes | 133(29.6) | | 61(11.9) | | 3.29(2.09-5.19) | |
| Cpn IgA× Passive smoking | | |  | |  | | 0.71(0.36-1.38) |
| *RERI*(95%*CI*) | | |  | |  | | 1.08(-0.26-2.43) |
| *API*(95%*CI*) | | |  | |  | | 0.33(-0.01-0.66) |
| *S*(95%*CI*) | | |  | |  | | 1.90(0.81-4.47) |
| Cpn IgG | Drinking |  | |  | |  | |
| - | No | 91(20.3) | | 143(27.9) | | 1.00 | |
| - | Yes | 30(6.7) | | 42(8.2) | | 0.71(0.37-1.36) | |
| + | No | 217(48.3) | | 262(51.2) | | 1.18(0.82-1.72) | |
| + | Yes | 111(24.7) | | 65(12.7) | | 1.43(0.87-2.37) | |
| Cpn IgG× Drinking | | |  | |  | | 0.67(0.31-1.44) |
| *RERI*(95%*CI*) | | |  | |  | | 0.55(-0.17-1.26) |
| *API*(95%*CI*) | | |  | |  | | 0.38(-0.06-0.82) |
| *S*(95%*CI*) | | |  | |  | | -3.92(-) |
| Cpn IgA | Drinking |  | |  | |  | |
| - | No | 190(42.3) | | 296(57.8) | | 1.00 | |
| - | Yes | 75(16.7) | | 80(15.6) | | 0.94(0.59-1.49) | |
| + | No | 118(26.3) | | 109(21.3) | | 1.50(1.03-2.18) | |
| + | Yes | 66(14.7) | | 27(5.3) | | 1.90(1.05-3.42) | |
| Cpn IgA× Drinking | | |  | |  | | 0.81(0.38-1.70) |
| *RERI*(95%*CI*) | | |  | |  | | 0.46(-0.70-1.61) |
| *API*(95%*CI*) | | |  | |  | | 0.24(-0.27-0.75) |
| *S*(95%*CI*) | | |  | |  | | 2.05(0.32-13.04) |
| Cpn IgG | Family history of cancer |  | |  | |  | |
| - | No | 83(18.5) | | 145(28.3) | | 1.00 | |
| - | Yes | 38(8.5) | | 40(7.8) | | 1.70(0.93-3.13) | |
| + | No | 253(56.3) | | 269(52.5) | | 1.31(0.90-1.91) | |
| + | Yes | 75(16.7) | | 58(11.3) | | 2.49(1.47-4.22) | |
| Cpn IgG× Family history of cancer | | |  | |  | | 0.95(0.44-2.08) |
| *RERI*(95%*CI*) | | |  | |  | | 0.47(-0.91-1.86) |
| *API*(95%*CI*) | | |  | |  | | 0.19(-0.32-0.70) |
| *S*(95%*CI*) | | |  | |  | | 1.47(0.44-4.87) |
| Cpn IgA | Family history of cancer |  | |  | |  | |
| - | No | 195(43.4) | | 305(59.6) | | 1.00 | |
| - | Yes | 70(15.6) | | 71(13.9) | | 1.92(1.23-3.02) | |
| + | No | 141(31.4) | | 109(21.3) | | 1.70(1.18-2.45) | |
| + | Yes | 43(9.6) | | 27(5.3) | | 2.59(1.41-4.78) | |
| Cpn IgA× Family history of cancer | | |  | |  | | 1.32(0.60-2.91) |
| *RERI*(95%*CI*) | | |  | |  | | -0.03(-1.76-1.70) |
| *API*(95%*CI*) | | |  | |  | | -0.01(-0.68-0.66) |
| *S*(95%*CI*) | | |  | |  | | 0.98(0.33-2.88) |

^a^Adjusted by age, sex, education, occupation, BMI, smoking, passive smoking, alcohol consumption, history of lung diseases, history of other diseases, family history of cancer, occupational physical activity, physical exercise, cooking oil fumes and pollution near the residence.
